# Management of HIV-2 resistance to antiretroviral therapy in a HIV-1/HIV-2/HBV co-infected patient

**DOI:** 10.1186/s12981-021-00394-4

**Published:** 2021-10-12

**Authors:** Margarida Cardoso, Joana Vasconcelos, Teresa Baptista, Isabel Diogo, Fátima Gonçalves, Kamal Mansinho, Perpétua Gomes

**Affiliations:** 1 Serviço de Doenças Infeciosas e Medicina Tropical, Hospital De Egas Moniz - Centro Hospitalar Lisboa Ocidental, 1349-019 Lisbon, Portugal; 2 Laboratório de Biologia Molecular, LMCBM, SPC.HEM - Centro Hospitalar Lisboa Ocidental, 1349-019 Lisbon, Portugal; 3 Centro de Investigação Interdisciplinar Egas Moniz, CiiEM, ISCSEM, 2829-511 Almada, Portugal

**Keywords:** HIV-2, HIV-1, Resistance, Mutation, Coinfection, Antiretroviral therapy

## Abstract

**Background:**

The current standard of care is to start antiretroviral therapy in all patients diagnosed with HIV-1, as for HIV-2 current DHHS guideline suggests ART for HIV-2 as soon as diagnosis is established, although this practice is not universal, for instance, in Portugal there are specific criteria to start treatment.

**Case presentation:**

We present a case of a man, chronically infected with HIV-1, HIV-2 and hepatitis B virus who developed resistance to HIV-2 while maintaining HIV-1 under control. 6 years after starting antiretroviral therapy he had his first virologic failure. We performed HIV-2 resistance tests that revealed high-grade resistance to all nucleoside reverse-transcriptase inhibitors except tenofovir and to all protease inhibitors except darunavir. After a decade of permanent poor adherence to therapy he developed resistance to both tenofovir and darunavir. We put together a new regiment with tenofovir alafenamide + emtricitabine + dolutegravir + maraviroc and nowadays he is with undetectable HIV-1 and HIV-2 viral loads.

**Conclusions:**

This shows the importance of having access to HIV-2 viral load determination and HIV-2 resistance testing.

## Background

Human immunodeficiency virus type 1 (HIV-1) infection is responsible for the majority of human immunodeficiency virus cases worldwide. In West Africa human immunodeficiency virus type 2 (HIV-2) is also prevalent with a 0.3–1% estimated coinfection rate [1]. These viruses have different natural history, with HIV-2 usually progressing more slowly than HIV-1 [2].

The current standard of care is to start antiretroviral therapy (ART) in all patients diagnosed with HIV-1 [3]. In Portugal, the criteria to start treatment in HIV-2 is: TCD4 + count under 350 cells/uL or symptomatic infection or viral load over 100 cp/mL in two sequential measures [4]. HIV-2 is naturally resistant to non-nucleoside reverse-transcriptase inhibitors, susceptible to integrase strand-transfer inhibitors and nucleoside reverse-transcriptase inhibitors and exhibits variable susceptibility to different protease inhibitors [2]. Recently, in 2021, an effort was made by a panel of European HIV-2 experts, to create recommendations for the diagnosis, treatment and follow-up of HIV-2 patients based on the published literature as well as field experience. They recommend to initiate ARV in all symptomatic patients and in asymptomatic with one of the following conditions:

- TCD4 + count ≤ 500 CD4 + -cells/μL blood,
- TCD4 + decrease of more than 30 cells/μL and year, over a period of more than 3 years,
- Repeatedly detectable HIV-2 RNA in plasma,
- Comorbidities, such as chronic HBV infection [5].

## Case presentation

We present a case of a 54 year old Guinean man, chronically infected with human immunodeficiency virus type 1 (HIV-1) subtype B, human immunodeficiency virus type 2 (HIV-2) group A and hepatitis B virus (HBV) with negative HBeAg. Previous medical conditions included hypertensive cardiac insufficiency and a chronic kidney disease.

The diagnosis of HIV-1 and HIV-2 coinfection was initially made with serological testing, initially a 4th generation assay, followed by positive HIV-1 and HIV-2 Western Blot. HIV-1 viral load was detected using a commercial assay and HIV-2 viral load was detected using an in-house real-time RT-PCR. HIV-2 resistance testing was done with an in house assay. The viral RNA was extracted from plasma using BioMerieux’s Nuclisense® EasyMag® equipment. The integrase and pol genes were amplified using a protocol in house, followed by sequencing by the Sanger method using 8 primers and the BigDye® Terminator v.3.1 Cycle Sequencing Kit. Detection and reading of DNA sequences was performed on the sequencer Automatic ABI Prism ®3100 from Applied Biosystems. Finally the DNA sequences were stored in the RegaDb database, Leuven (Rega Institute; REGA), analyzed using the CromasPro V software. 1.7.6. and interpreted according to the Grade HIV-2 algorithm (https://bit.ly/378Q3tK).

The patient was diagnosed in 2001 with an initial CD4 245 cells/uL, during pre-surgical screening. According to Centers for Disease Control and Prevention, Atlanta classification, he had category A2 disease. He had not been tested for HIV previously and was probably infected through heterosexual contact. The patient has been followed up twice a year, with viral load monitoring every 6 months in the infectious diseases’ ambulatory clinic since July 2001.

In 2001 he started ART with atazanavir/lamivudine + nelfinavir (ATV/3TC + nelfinavir). Therapy was switched to ATV/3TC + lopinavir and ritonavir (LPVr) in 2007 due to HIV-2 virologic failure [viral load (VL) = 388 cp/mL] with undetectable HIV-1 VL.

In 2011 he had another HIV-2 therapeutic failure (VL = 2771 cp/mL), with undetectable HIV-1 VL, high-grade resistance to all nucleoside/nucleotide reverse transcriptase inhibitors (NRTIs) except tenofovir disoproxil fumarate (TDF) [Q151M, M184V] and high-grade resistance to all protease inhibitors (PIs) inhibitors except for darunavir (DRV) [V47A, L90M] was shown on further testing. Therefore, ART was switched to TDF/emtricitabine + DRV/r + raltegravir.

In August 2018 he had undetectable VL to both HIV-1 and HIV-2. However, half a year later he had another virologic failure, this time to both viruses, HIV-1 (VL = 7352 cp/mL) and HIV-2 (VL = 754 cp/mL). We performed both resistance tests and HIV-2 resistance test revealed high grade resistance to all NRTIs [K65R, D67N, Q151M, S215ST] and high-grade resistance to LPV, saquinavir and DRV [V47A, I84V, L90M]. HIV-2 and HIV-1 resistance tests to integrase strand-transfer inhibitors were negative, as well as HIV-1 to PIs and reverse-transcriptase inhibitors.

**Fig. 1:**
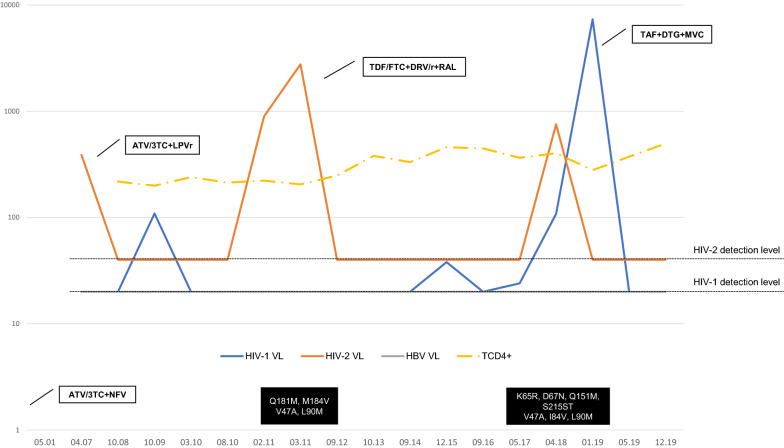
Patient HIV-1/HIV-2/HBV viral loads and TCD4 + count over calendar time. This graphic represents the evolution of the HIV-1/HIV-2/HBV viral loads and TCD4 + count between diagnosis in 2001 and last evaluation of the patient in 2019. The curves were represented in a logarithmic scale for better understanding. Additionally we highlighted the moments when ARV was changed (white boxes) and mutations detected (black boxes). The level of detection illustrated is < 20 copies/mL for HIV-1, < 40 copies/mL for HIV-2 and < 20 copies/mL for HBV. Lamivudine (3TC), atazanavir (ATV), darunavir (DRV), dolutegravir (DTG), emtricitabine (FTC) hepatitis B virus (HBV), hepatitis B virus (HBV), human immunodeficiency virus type 1 (HIV-1), human immunodeficiency virus type 2 (HIV-2), lopinavir (LPV), maraviroc (MVC), nelfinavir (NFV), raltegravir (RAL), tenofovir alafenamide fumarate (TAF), tenofovir disoproxil fumarate (TDF), viral load (VL)

Since January 2019, he has been taking tenofovir alafenamide fumarate + DTG + maraviroc and at the last evaluation he had undetectable HIV-1 and HIV-2 VL with a CD4 + cells count of 374 cells/uL. (Fig. 1).

It’s relevant to highlight that this is a patient with poor adherence to ART, who stopped ART and follow-up several times in the last years.

HBV VL has remained undetectable through all these years, without HBsAg seroconversion.

## Discussion and conclusions

This case emphasizes the difficulty of the management of a triple coinfection. We point to the possibility of virologic failure exclusively to HIV-2 with controlled HIV-1 and HBV infections. In fact, it has been reported that HIV-2 may slow down HIV-1 progression [6] and its optimal treatment is challenging considering its different susceptibility to ARV. In our patient it seems that the initial ARV could have been suboptimal for HIV-2, namely nelfinavir [7], leading to the development of mutations.

This shows the importance of having access to HIV-2 viral load determination and HIV-2 resistance testing and the need to keep updated in clinical practice in order to achieve an efficacious regimen to both HIV (1 and 2) and HBV infections. Taking into account the complexity of HIV-2 infection and less experience in this field we suggest that the management of this infection should be done or guided by experts in the area and if possible in reference centers.

## Data Availability

Not applicable.

## Abbreviations

3TC: Lamivudine
ART: Antiretroviral therapy
ATV: Atazanavir
DRV: Darunavir
HBV: Hepatitis B virus
HIV-1: Human immunodeficiency virus type 1
HIV-2: Human immunodeficiency virus type 2
LPV: Lopinavir
NRTIs: Nucleoside/nucleotide reverse transcriptase inhibitors
PIs: Protease inhibitors
r: Ritonavir
TDF: Tenofovir disoproxil fumarate
VL: Viral load
